# Naphthalene-Induced Acute Oxidative Hemolysis With Methemoglobinemia in Glucose-6-Phosphate Dehydrogenase (G6PD) Deficiency

**DOI:** 10.7759/cureus.23496

**Published:** 2022-03-25

**Authors:** Ramesh Thangatorai

**Affiliations:** 1 Internal Medicine, Hospital Sultan Ismail, Johor, MYS

**Keywords:** cyanosis, methemoglobinemia, napthalene, g6pd deficiency, haemolysis

## Abstract

Naphthalene-induced acute hemolysis with methemoglobinemia is a rare condition that commonly affects patients with glucose-6-phosphate dehydrogenase (G6PD) deficiency. It commonly presents with anemia, jaundice and features of methemoglobinemia such as cyanosis and falsely low pulse oximetry. We present a patient with naphthalene-induced methemoglobinemia and discuss the clinical features, pathophysiology and management of this oxidative hemolysis. Additionally, we discuss the possibility of false-positive d-dimer and false-negative G6PD screening during acute hemolysis.

## Introduction

Naphthalene is an organic compound found in many mothballs, which is a common household item. It is commonly used as an insect repellant, especially in closets making it very accessible to children. Ingestion of naphthalene-containing mothballs is known to cause oxidative hemolysis in children with glucose-6-phosphate dehydrogenase (G6PD) deficiency [1]. However, case reports on adult patients are rare. Naphthalene ingestion leads to oxidative hemolysis and then causes the production of methemoglobins. This leads to methemoglobinemia, which has a vast variety of presentations. We report a patient who developed acute oxidative hemolysis and methemoglobinemia due to naphathalene ingestion. 

## Case presentation

A 23-year-old male with no known medical illness presents with fever, runny nose, and sore throat for three days. He was suffering from work-related stress, thus on day two of fever, he ingested one naphthalene ball. One day post-ingestion he developed nausea, vomiting with jaundice and dark urine. He presented to our emergency department on day three of illness with severe lethargy, jaundice, and dark urine. 

Physical examination revealed a pale and deeply jaundiced young man, with a blood pressure of 123/73 mm Hg and tachycardic at 102 bpm. His oxygen saturation (SpO2) was 79% under 15L/min oxygen flow. He had an otherwise unremarkable physical examination, with no hepato-splenomegaly nor any lymph nodes.

**Figure 1:**
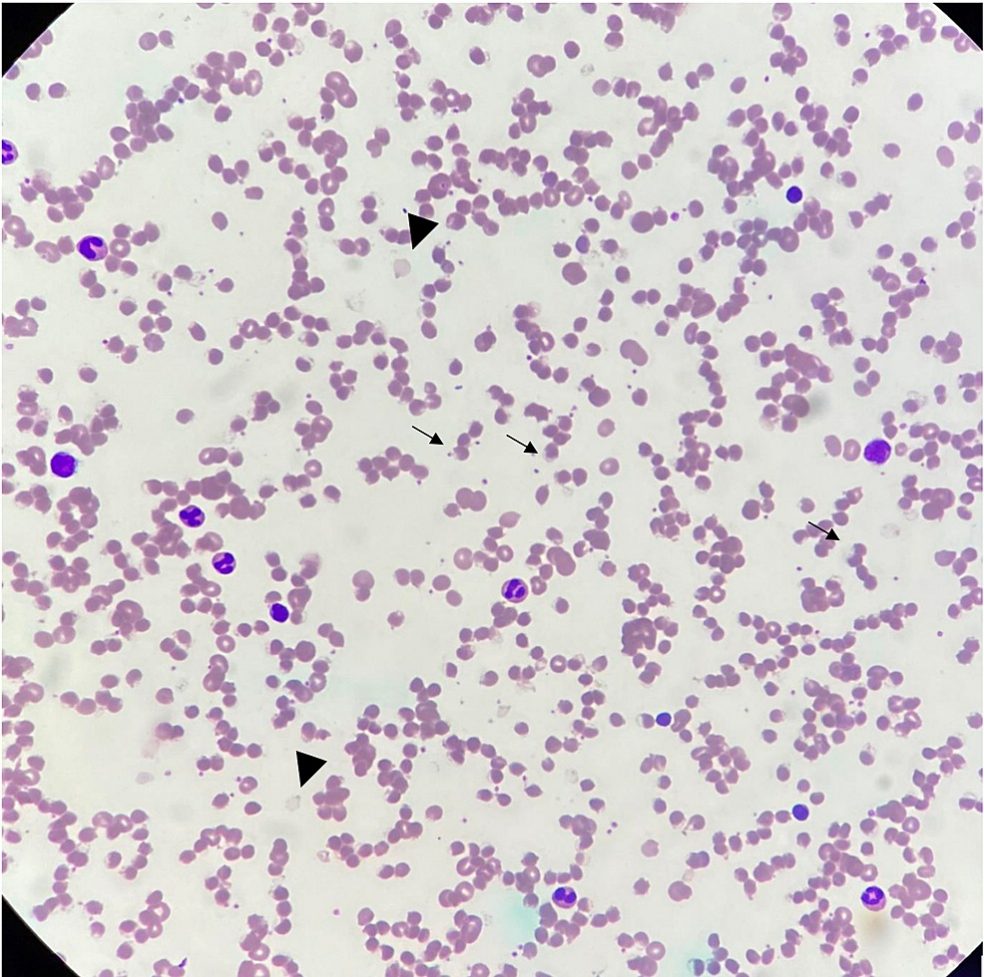
Full blood picture Arrowheads: Ghost cells; Arrows: Blister cells

Blood Investigations on the day of admission showed severe macrocytic anemia with increased white blood cells and normal platelets. He had indirect bilirubinemia with elevated lactate dehydrogenase, reticulocytosis and mild acute kidney injury that indicates ongoing hemolysis. His blood parameters are detailed in Table 1. Despite his high oxygen requirement, his arterial blood gas and chest X-ray were unremarkable. Urgent peripheral blood film was suggestive of oxidative hemolysis because of bite cells and blister cells as shown in Figure 1.

**Table 1:** Blood investigations WBC: White blood cells; MCV: Mean corpuscular volume; MCHC: Mean corpuscular hemoglobin concentration; LDH: lactate dehydrogenase; PaO2: Partial pressure of Oxygen

	17th August 2020	18th August 2020	19th August 2020	20th August 2020	21st August 2020	22nd August 2020	23rd August 2020
WBC (X10 3/UL)	18.1	16.6	16.6	13.6	9.9	7.0	6.5
Hemoglobin g/dl	5.6	6.3	5.7	6.3	6.7	7.8	8.4
MCV	100	95.7	93.7	92.6	93.4	95.8	96.3
MCHC	33.8	32.8	32.7	31.9	31.5	31.4	32
Platelet (X10 3/UL)	270	164	152	128	111	164	202
Reticulocytes (X10^3^/UL) Normal values (40 - 75)	105		107	151			
LDH	3300		3220	2929	2645	1642	1319
C-Reactive Protein	101						
Total Bilirubin (umol/l)	115	104	77.3	25.4	14.4	8.7	6.4
Direct Bilirubin (umol/l)	12.9	20.4					
Indirect Bilirubin (umol/l)	102	81					
Urea (mmol/l)	10.4		12.4	5.4	3.6	4.6	
Creatinine (mcmol/l)	98		79	64	51	59	
PaO2 (mmHg)		125 mmHg (under RA)					
D-Dimer		Positive					
Methemoglobin level				3.3%			

Given his persistently poor pulse oximetry reading and a positive D-Dimer, we proceeded with CT pulmonary angiography (CTPA) which was negative for pulmonary embolism. We were only able to send a serum sample of methemoglobin on day 4 of admission (as it is not available in our center), which was 3.3% (reference range: <1.5). However, the results were only available a month after sampling. G6PD testing was normal on day 2 of admission. We treated him as naphthalene-induced oxidative hemolysis and sepsis of unknown source, given raised C-reactive protein (CRP).

We hydrated him with 3.5L of normal saline /24 hours and regular blood transfusion, aiming for hemoglobin (Hgb) > 8.0. We also supplemented him with T folic acid 5 mg daily and empirically treated him with IV augmentin for 7 days. The patient recovered well on day 5 of admission, with a static hemoglobin level and his saturation level on pulse oximetry returned to normal. We followed up with him 6 weeks later and his Hgb normalized to 15.1. A repeat G6PD test reported deficiency, indicating the first normal G6PD assay was a false negative. However, we do not have the enzyme level available in our setting.

## Discussion

Naphthalene in the mothballs enhances free radical production, leading to oxidative hemolysis and methemoglobinemia [2]. 

The free radicals oxidize Fe2+ in the hemoglobin to Fe3+ leading to the production of methemoglobins [1,3]. Methemoglobins are unable to bind to oxygen, unlike regular hemoglobins. In our patient, we were alarmed by the Sp02, which persistently showed 70%-80% under room air, improving to merely 90% under a high flow mask with 15L/min oxygen. This is due to methemoglobins equally absorbing both the red and infrared lights emitted by the pulse oximetry probe, thus leading to oxygen saturation interpreted to be falsely low by the oximetry probe [4]. Thus in patients with suspected methemoglobinemia, arterial blood gas monitoring is more accurate compared to pulse oximetry, especially if the patient’s clinical status does not match the pulse oximetry reading, as in our case. Apart from falsely low peripheral saturation, methemoglobinemia also causes a variety of symptoms, depending on the percentage of methemoglobin present. At the concentration of 30%-50%, confusion, tachycardia, and tachypnea develop. When methemoglobin levels reach more than 50%, patients may experience seizure, coma or death [5].

We ordered a CTPA for this patient as few case reports suggest a high risk of thrombosis in patients with hemolytic anemia [6-7]. D-dimer is a fibrin degradation product that is released when blood clots are degraded by fibrinolysis. D-dimer should be used as a “rule out” test for thrombo-embolic disorders as it has very high levels of false-positive results. We postulate that the most likely cause of the false-positive D-dimer in this patient is ongoing inflammation secondary to oxidative damage. There are no other case reports that comment on D-dimer in patients with oxidative hemolytic anemia.

This patient’s first G6PD activity level was normal, and a repeat test 2 months later showed G6PD deficiency. This phenomenon is due to the increased reticulocytes during the acute hemolysis, which has higher G6PD activity, leading to false-negative results [8]. G6PD enzyme level will give us more information regarding the enzyme activity level during active hemolysis and once hemolysis subsided, however, the test is not available in our centre. 

The management of oxidative hemolysis with methemoglobinemia is mainly supportive. A few case studies have reported using ascorbic acid 300 mg PO as part of the treatment [3-4]. A case report by Topal H and Topal Y reported a reduction in serum methemoglobin level after a single dose of IV ascorbic acid 300 mg over 24 hours [9].

Methylene blue reduces methemoglobin to hemoglobin by accepting electrons from NADPH (nicotinamide adenine dinucleotide phosphate hydrogen methemoglobin), thus is also used for the treatment of methemoglobinemia, however, it should not be used in patients with G6PD deficiency. This is because NADPH production requires G6PD enzyme activity [3,10].

Our patient was treated with adequate hydration and regular blood transfusion, and he showed improvement three days after his last intake of naphthalene balls.

## Conclusions

The diagnosis of methemoglobinemia requires very high index of suspicion. In this patient, persistent cyanosis and low oximetry reading despite having a clear chest X-ray and normal PaO2 in arterial blood gases raised the suspicion of methemoglobinemia. Combined with findings of oxidative hemolysis in the peripheral blood film, we were able to clinch the diagnosis of naphthalene-induced oxidative hemolysis leading to methemoglobin production. A thorough history, especially regarding birth G6PD screening, and new initiation of drugs, including traditional medications, are of utmost importance in dealing with patients who present with acute hemolytic crisis and methemoglobinemia.
